# Impact of mobile phone-based technology to improve health, population and nutrition services in Rural Bangladesh: a study protocol

**DOI:** 10.1186/s12911-017-0502-9

**Published:** 2017-07-06

**Authors:** Jasim Uddin, Tuhin Biswas, Gourab Adhikary, Wazed Ali, Nurul Alam, Rajesh Palit, Nizam Uddin, Aftab Uddin, Fatema Khatun, Abbas Bhuiya

**Affiliations:** International Centre for Diarrhoeal Disease Research, Dhaka, Bangladesh

**Keywords:** Health services, mHealth technology, Smartphones, Bangladesh

## Abstract

**Background:**

Mobile phone-based technology has been used in improving the delivery of healthcare services in many countries. However, data on the effects of this technology on improving primary healthcare services in resource-poor settings are limited. The aim of this study is to develop and test a mobile phone-based system to improve health, population and nutrition services in rural Bangladesh and evaluate its impact on service delivery.

**Methods:**

The study will use a quasi-experimental pre-post design, with intervention and comparison areas. Outcome indicators will include: antenatal care (ANC), delivery care, postnatal care (PNC), neonatal care, expanded programme on immunization (EPI) coverage, and contraceptive prevalence rate (CPR). The study will be conducted over a period of 30 months, using the existing health systems of Bangladesh. The intervention will be implemented through the existing service-delivery personnel at various primary-care levels, such as community clinic, union health and family welfare centre, and upazila health complex. These healthcare providers will be given mobile phones equipped with Apps for sending text and voice messages, along with the use of Internet and device for data-capturing. Training on handling of the Smartphones, data-capturing and monitoring will be given to selected service providers. They will also be trained on inputs, editing, verifying, and monitoring the outcome variables.

**Discussion:**

Mobile phone-based technology has the potential to improve primary healthcare services in low-income countries, like Bangladesh. It is expected that our study will contribute to testing and developing a mobile phone-based intervention to improve the coverage and quality of services. The learning can be used in other similar settings in the low-and middle-income countries.

**Electronic supplementary material:**

The online version of this article (doi:10.1186/s12911-017-0502-9) contains supplementary material, which is available to authorized users.

## Background

Antenatal care (ANC), postnatal care (PNC), delivery care, and age at first marriage are important factors that can ensure safe motherhood. In Bangladesh, only 26% women receive the recommended four or more ANC visits, and 68% receive at least one ANC visit during their pregnancies; only 27% receive PNC, and only 29% deliveries are taken place at facilities. These are some of the important challenges facing Bangladesh for ensuring safe motherhood in the country [1]. The situation needs to be improved substantially.

Almost half of the women give birth by the age of 18 years and nearly 70% by the age of 20 years [2]. It is necessary to increase the age at first birth as data indicate complications for both child and the mother for early marriage of women. Despite tremendous success of EPI in Bangladesh, a substantial number of children are not fully vaccinated under expanded programme on immunization (EPI) as data show 82% were fully vaccinated by the age of 12 months [3].

In Bangladesh, about 2 million new faces are added to the population annually, with a growth rate of 1.4% [4]. The Health, Nutrition and Population Strategic Investment Plan (HNPSIP) 2016–2021 of Ministry of Health and Family Welfare (MOHFW) of the Government of Bangladesh set a target of achieving total fertility rate (TFR) of 1.7 and contraceptive prevalence rate (CPR) of 75% by 2021, which is currently 2.3 and 61.2 respectively [5] that need further interventions to achieve the targets fully.

In recent years, the use of mobile phone-based technology in healthcare (mHealth) has emerged to augment the healthcare services where the population is underserved, especially in rural areas [6]. In a systematic review, Tamrat and colleagues (2011) highlighted the scope of mHealth along the stages of ‘continuum of care’ for maternal, new born and child healthcare [7]. The mHealth programmes, along with SMS-based health education, are being used for pregnancy tracking and reminders for ANC visits. During the birth, mHealth can be used for point-of-care remote consultation, facilitating referral and access to health facilities and for promoting timely contact with the community health workers. For postpartum and new born care, mHealth can be used for monitoring maternal health and infants’ growth, and timely reminders for immunization [7].

In Bangladesh, the mHealth and eHealth initiatives are being implemented by the MOHFW and different organizations with donor support. These initiatives complement the existing health systems and contribute to increasing the effectiveness of health, population and nutrition (HPN) services.

The Government of Bangladesh has placed a high priority on eHealth, which is reflected in the ICT Policy 2009. The strategic areas/issues relevant to health in the ICT Policy 2009 (Clauses 7.1–7.4 of ICT Policy) include the following: improve healthcare delivery management through the use of telemedicine and modern technology; create awareness at all levels, including hard-to-reach areas with particular importance in making maternal, child and reproductive care available; and ensure quality of care and increase the capacity of health care delivery system [8]. However, the use-pattern of technology for covering all or major components of primary healthcare (PHC) is yet to be developed and tested in Bangladesh. Further, no such initiative has yet been taken focusing on community clinic (CC) to ensure universal health coverage in Bangladesh.

At present, the CCs in Bangladesh can cater the services on family planning (FP), maternal, neonatal and child health (MNCH) and health education for the rural people by using eHealth strategy as the community healthcare providers (CHCPs) and other staff at CCs are equipped with Internet-connected laptop computers [9]. Findings of a recent study show that 33% rural married women and 80% of households have mobile phones [4]. Therefore, the MOHFW, icddr,b, and Ethics Advance Technology Limited (EATL) have taken the initiative to utilize mobile phone-based technology to improve HPN service in rural areas of Bangladesh. This study will be implemented with the aim to develop and test a mechanism of mHealth strategies as well as assessing its impact in improving reproductive health and family planning, maternal, neonatal, and child health, integrated management of childhood illness, expanded programme on immunization, and other primary healthcare services at the community level in rural areas of Bangladesh. This will be an evidence-based research for HPN services, using mobile phone-based technology integrated with health system of the Government in Bangladesh.

This study aims to: (i) improve the coverage of ANC, delivery care and PNC among pregnant woman; (ii) Improve the use of family planning services among newly-married couples; (iii) improve utilization of MNCH services, including EPI, to reduce neonatal and child mortality rate; and (iv) establish technology-based referral linkage of CCs with the higher facilities, such as union health and family welfare centre (UH&FWC) and upazila health complex (UHC).

## Methods

### Study design

The study will follow a quasi-experimental pre-post design. Evaluation will be carried out through comparing ANC, delivery care, PNC, contraceptive prevalence rate (CPR) and EPI coverage before and after implementation of the intervention; the results will also be compared with data obtained from the comparison areas to assess the impact of the intervention.

### Study area

The study will be conducted in two selected administrative divisions (regions) of Bangladesh: one high-performing division (Rajshahi) and one low-performing division (Chittagong). The districts, selected based on the performance of health and family planning indicators, are: Natore from the high-performing Rajshahi division and Cox’s Bazar from the low-performing Chittagong division. In the high-performing division, one upazila/sub district (Bagatipara) of Natore will be selected as intervention area while Baraigram upazila of the same district will be used as comparison area. One upazila (Chakaria) of Cox’s Bazar district will be selected as intervention area while Ramu upazila of the same district will be used as comparison area. Thus, a total of 4 upazilas (2 as intervention and 2 as comparison) from the two divisions will be selected for the study.

### Timeline

The study will be conducted over a period of 30 months in three phases: (i) inception phase (6 months for IRB approval, recruitment of staff, stakeholders’ meetings at different levels, and survey instruments, training of government staff on the interventions, baseline survey, and preparation of the inception report); (ii) intervention implementation phase (18 months for implementation of the intervention; (iii) evaluation phase (4 months for endline data collection, data analysis and report writing, sharing of the results and evidence for future scale-up through dissemination, finalization of the report and publications).

### Development of mobile phone apps and testing

We have developed mobile phone apps. The mobile Apps were developed with support from a team of the Ethics Advanced Technology Limited (EATL). After developing the Apps, we did the field test and piloting in sites other than the project areas. After receiving feedback, we tried to make the easiest Apps. The SMS procedures were developed by a team comprising general physicians, immunization experts, family planning experts, health systems experts, healthcare providers, government and non-government officials. After developing the SMS procedures in Bangla language, we sent these messages to several individuals in the pre-test stage, including persons who can only read SMS and have no formal educational qualifications. After receiving feedback, we tried to make the contents of the SMS suitable for the general population. We will ensure that all of our participants in the study can read the SMS by themselves or someone in the family who can describe the messages to them if they do not understand.

### Interventions

The Bangladesh Government provides primary healthcare services to all citizens through a three-tiered healthcare service delivery system: community clinic, each for 8000 people; union health and family welfare centers (UH&FWCs), each for 25,000 people; and the upazila (sub district) health complexes (UHCs) with an outpatient and an emergency department, 50 inpatient beds and an operating room, each for 250,000 people. The service-delivery personnel at different levels (CC, UH&FWC, and UHC) will be given Smartphones having the facilities for the text and voice messages, using the Internet, and device for data-capturing. Smartphones will be provided to them from the project. Training on handling of the Smartphones, data-capturing and monitoring will be provided to upazila Health and family planning officers (UH&FPOs), upazila family planning officer (UFPOs), medical officers of the health and family planning departments, statistical assistants, family welfare visitors (FWVs), sub-assistant community medical officers (SACMOs), nurses, medical technologists, health inspectors (HIs), family planning inspectors (FPIs), assistant health inspectors (AHIs), health assistants (HAs), family welfare assistants (FWAs), community healthcare providers (CHCPs) and other related service providers in each upazila under study. They will also be trained on inputs, editing, verifying and monitoring the data on different services through the Apps installed in the Smartphones. The EATL team will provide technical support to improve the Apps, train concerned personnel, and extend support in smooth functioning of the system. The interventions to be tested are as follows:

#### Identification and registration of pregnant women and services for them

For fixing the target population for ANC, PNC, and delivery care, identification and registration of pregnant women will be the initial activity. A routinely-updated list of all pregnant women in every village will be available with all FWAs. However, this list can be updated with information from other service providers, like HAs, FWVs, and CHCPs. Pregnant women will be identified and registered using the following sources: (i) routine community visits of health and family planning workers; (ii) EPI centres when the pregnant mothers visit for receiving TT vaccine; (iii) registers of pregnant women maintained at community clinics; (iv) satellite sessions conducted by FWVs; (v) registers of pregnant women maintained by FWVs at UH&FWCs and UHCs; (vi) combined meetings held at community clinics, UH&FWCs and UHCs; and (vii) registers of pregnant women maintained at NGOs working on reproductive, maternal and child health.

##### Registration process of pregnant women

The Smartphones carried by the healthcare providers will contain customized software for registration of pregnant women with necessary information (name of pregnant woman, age, address, last menstruation period (LMP), and mobile phone number) and for updating and sending to the server. A unique identification (ID) number will be automatically created, followed by uploading of data to the server; the ID number will be given to the pregnant woman. This unique ID number will ensure her to get necessary healthcare services within the study area.

##### Service delivery system for ANC, delivery care, essential newborn care and PNC

Pregnant women will receive auto-reminders through their mobile phones for ANC visit prior to their scheduled date for each visit via voice message, followed by text message. Date of the scheduled visit will be calculated by the system automatically from the LMP. The reminders will include the date, time, available facilities, and services for ANC, and these will be sent to the women several times to ensure their ANC visits. The system will also send auto-reminder through text message to the concerned service provider to check whether the pregnant woman has received ANC or not. The service provider will provide and update the ANC services and status of the particular pregnant woman. Apart from reminders for ANC, the pregnant women will also receive voice message on healthcare during pregnancy, danger signs, and birth preparedness. From the LMP, the expected date of delivery (EDD) will be calculated by the system, and reminders will be sent to the pregnant women automatically. The pregnant woman will also automatically receive voice and text messages containing necessary information relating to institutional delivery according to her EDD.

#### Birth notification

The pregnant women will be encouraged and trained to notify immediately after the delivery through SMS by themselves or by anyone on behalf of them. After receiving birth notification, the system will automatically update the particulars of newborns, including date of birth, PNC schedule, due dates of EPI vaccination for the newborns and location of the service centre. The system will also send auto-reminders to the mothers with a specific time schedule for PNC visit, newborn care visit and scheduled dates of receiving vaccines for the newborns.

#### Childhood vaccination services under EPI

After the outcome of delivery by the registered women, the system will receive the birth notification as mentioned in the birth notification process. Mothers/caregivers of the newborns will receive voice and text messages one day prior to their visit to the vaccination centres. A second reminder will be sent to the parents of all the targeted children through their own phones or that of their family numbers collected earlier. A third reminder will be sent to the mothers/caregivers of children who were not vaccinated after receiving the second reminder as scheduled on that day. The system will also provide auto-reminders to the concerned service providers. The service providers will update the status of the vaccination of session, using their Smartphones, and the system will allow the service providers to see the drop-out and left-out cases.

#### Identification of newly-married couples

Identification and registration of the newly-married couples will be the initial activity for fixing the target population for FP service. A routinely-updated list of all newly-married couples in every village will be available with all FWAs. However, these data can be updated with information from other service providers also, such as HAs, FWVs, CHCPs. Identification and registration of couples will be done using the following sources: (i) regular visits to the community; (ii) service-delivery points, like community clinics, UH&FWCs, and UHCs; and (iii) satellite sessions by FWVs; and (iv) NGOs working on reproductive health.

##### Registration process for the newly-married couples

The Smartphones will contain specific software for registration of newly-married couples, through which some necessary information (names of both wife and husband, age, address, LMP, current use of contraceptives, future plan for childbirth, and mobile phone numbers) can be updated and sent to the server. A unique ID number will be automatically created, followed by uploading of data on the server; the ID number will be given to the couple. This unique ID will ensure them to get necessary family planning services within the study area.

##### Family planning service-delivery system

Based on the information gathered through registration process for the couples, the system will automatically generate the most suitable family planning options for a couple and send the information to the concerned service providers. The service provider will motivate the clients for the most suitable FP method option generated by the system and, after taking the consent, they will provide the method accordingly. The system will send auto-messages to the couples through their mobile phones about the importance of birth spacing and the use of FP methods, information relating to services available at CCs and UH&FWCs. The service providers will update this information in the system, and the regular follow-up status will be recorded in the server.

#### eReferral

There will be an online referral system for the registered women for ANC, PNC, delivery care, neonatal care, IMCI, and management of complications, through which healthcare providers from lower facilities can refer a patient to the higher facilities. The women will be identified in the higher facilities by their unique ID numbers. The service providers will enter referral data to the system during the referral process, and the patients and care providers from higher facilities will get system-generated reminders about the referral. The service providers in higher facilities will be able to check the health status and cause of referral, using the patient’s unique ID number from the system and will manage accordingly as well as update information on the management in the system. If the referred woman does not visit the referral facility, she will receive repeated reminders auto-generated by the system to comply with the referral; the service provider who referred the client will be able to see the status, using Smartphone.

#### eMonitoring

**Fig. 1:**
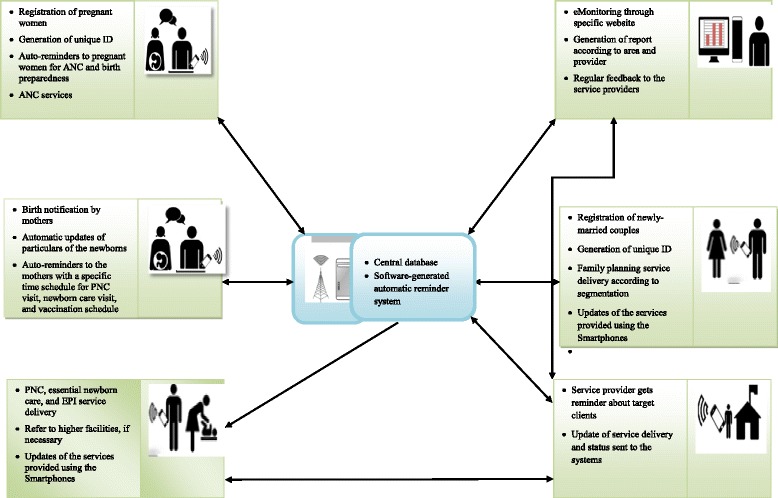
Implementation process of the interventions

**Fig. 2:**
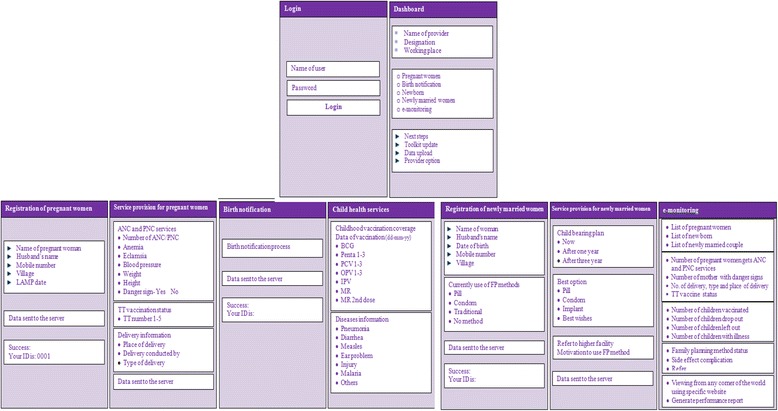
Snap shots of the Apps developed

There will be a web-based monitoring system to oversee the progress and status of all activities under this study for policy-makers, programme managers, and other supervisors from national to the union levels. All these data will be available in the central database which will be visualized and accessible at all concerned levels through the Internet in particular website. They can constantly follow up the progression and healthcare delivery system of the respective areas and provide regular feedback, when necessary. There will be a provision for auto-generation of reports for each and every provider-specific activity through the system by area. Figure 1 shows the implementation process of the interventions, and Fig. 2 shows the snap shots of Apps developed for the purpose.

### Evaluation

#### Study population

The study populations will include: (i) currently-married women of reproductive age, who are not currently pregnant and did not have delivery within the last 6 months; (ii). mothers of children aged 12–23 months for assessing children’s vaccination coverage; (iii). mothers of children aged 0–11 months for assessing ANC, delivery care, PNC, and vaccination coverage; and (iv).healthcare providers.

#### Sample-size

A four-cell (pre-and post surveys in the intervention and comparison areas) study design will be followed for evaluation of the effects. A stratified two-stage random cluster-sampling technique will be followed for selecting respondents in the community survey. The first stage will be the selection of a segment (or area covered by EPI centre) from the catchment area of CC, and the second stage will be selection of households with eligible target populations. The cluster-sampling methodology will be used in estimating the measurable indicators.

We aim that the proposed mHealth project will change the measurable indicators: contraceptive prevalence rates, antenatal care visits from skilled community midwives, delivery attended by skilled birth attendants (either at home or health facility); postnatal care from skilled birth attendants by 10% points or more from the baseline level in the study area. The minimum required sample-size for detecting 10% points or more per group per stratum per survey is estimated to be 660. Total sample-size for the baseline and end line surveys for impact evaluation is 5280.

##### Procedures for selection of sample clusters

In the catchment area of a CC there are 8 routine EPI outreach centers (including one in the CC) each serving, on an average, ~1000 people (or ~235 households). The catchment area of the EPI centre will be treated as ‘cluster’. Given the crude birth rate of ~20 per 1000 population per year, there will be 20 mothers each of whom has an infant aged 0–11 months and is eligible to provide information on ANC, delivery care, and PNC; and another 20 mothers each of whom has a toddler aged 12–23 months and is eligible to provide information on child vaccination. As such, 33 EPI centers from the list in an upazila (with at least one EPI centre per CC) in the selected sub district will be randomly chosen for survey.

##### Selection of households

The second stage will be the selection of households with eligible respondents in each sampled cluster (or catchment area of EPI centre). All mothers with child aged 0–11 months or 12–23 months will be interviewed, with their consents, for maternity care and vaccination respectively. Women (married non-pregnant or did not deliver babies in <6 months) eligible for family planning method-use are many in a cluster. Therefore, a woman eligible for family planning method-use living in the household next to the mother with a child aged 0–11 months will be interviewed. The same sample selection procedures will be followed in the comparison areas. Pre-tested data collection tools (Additional file 1) will be used for collecting data.

##### Data analysis

The analysis of data from the baseline and end line surveys in the intervention and comparison areas will assess changes (difference-in-difference) in selected indicators (four or more ANC visits as recommended, institutional delivery, PNC, full vaccination coverage for children, and CPR). Logistic regression will be used for statistical significance in indicators.

### Ethics

Confidentiality of all potential participants will be protected when invited to take part in the study. Respondents will be interviewed after obtaining informed written consents. All respondents will be properly informed about the study and be thoroughly made to understand what their participation in the study involves. Participation will be voluntary. The participants will be ensured that refusal to participate will have no adverse consequences for them. Confidentiality of the data will be strictly maintained, and restrictions on access to data-forms will be enforced. The study itself does not involve any physical, social or legal risks to the participants. Information will be collected through interviews and discussions. The participants will be assured that the information provided by them will be used for research purposes only and would not be shared anywhere with names of the participants. Interviews will be conducted according to the respondents’ convenience. The protocol was reviewed by both icddr,b’s internal and external experts for its submission for ethical approval. Ethical approval (PR-16019) for the study is obtained from the institutional review board of icddr,b. All the collected data will be de-identified and will be kept in the MOHFW’s server. Research data will be sought for the analysis, report writing, and publication. Research data will be kept in a password-protected computer at icddr,b.

## Discussion

This article describes the protocol for assessing the impact of mobile phone-based technology to improve utilization of the health, population and nutrition (HPN) services in rural Bangladesh. This study will be the first of its kind in Bangladesh, to the best of our knowledge, to assess the impacts of mobile phone-based technology for primary healthcare services in rural areas. This study will help generate hypothesis, evidence-based real-time data, develop programmes and policies to improve utilization of the health, population and nutrition (HPN) services through mobile phone-based technology in other similar countries also.

A recent study in rural Bangladesh reported that 80% of the households had at least one mobile phone and, among them, 31% of the respondents reported that mobile phone is being used for healthcare [10]. It was also reported that ownership of mobile phones is comparatively higher among the males compared to the females. However, a study reported that, globally, women are 21% less likely to own a mobile phone than men, and this difference is higher in South East Asia [11]. A nationwide study conducted in Kenya showed that ownership of mobile phones was 1.7 times and SMS-use was 1.6 times higher among males than among females [12]. On the other hand, a recent review of the growth of mHealth services in Bangladesh documented 19 initiatives involving mHealth services from call centers sending messages from health authorities to subscribers [13]. However, all of these initiatives have been mainly focusing on urban population.

Akter et al. (2010) evaluated the service quality of Health Line 789 in Bangladesh and found that service quality has a significant impact on satisfaction, intention to continue using services, and improve the quality of life [14]. Khatun et al. examined the readiness of mHealth services in rural Bangladesh; findings showed that rural community of Bangladesh had a degree of readiness for the use of technology for motivational purposes but readiness for seeking care was likely to be limited due to inequitable access to technology [15]. Our study is likely to promote ownership of and access to mobile phones by females.

A review suggests that SMS-delivered interventions have positive short-term behavioral outcomes [16]. Another study also reported efficacy of mHealth interventions in low- and middle-income countries (LMICs), particularly in improving adherence to treatment, compliance with appointment, data-gathering, and developing support networks for health workers [17]. Similarly, another study also demonstrated that there is a huge opportunity of mobile technology for healthcare in the developing world [18]. A study in neighboring country India reported that most of the respondents were willing to receive health-related information on their mobile phones [19]. A study from Nakuru, Kenya, established that mobile phones were useful in facilitating communication and decision-making in reproductive health [20]. As in developing countries, a study from Washington, DC reported participants’ willingness to send photographs of their wounds to physicians for diagnosis and recommendations [21]. For MNCH services to help professional and traditional birth attendants to manage postpartum hemorrhage, mHealth was used for clinical reporting in a rural community of Ghana [22]. In this particular project, traditional birth attendants could report real-time health-related data, such as perinatal care, postpartum hemorrhage, and maternal and child death, with the help of a mobile phones [22]. In rural Argentina, a study conducted among pregnant mothers showed that the majority (96%) of respondents valued receiving messages regarding pregnancy-related health information and postpartum care through SMS in their mobile phones or via email [23]. A study conducted in rural Bangladesh showed that toll-free mobile phone services for maternal and child healthcare intervention were effective compared to the usual care [24, 25]. Active participation of community health workers (CHWs) and mothers’ accessibility to CHWs through mobile phones for maternal health issues increased the effectiveness of maternal healthcare [24, 25].

Despite substantial improvements in health in recent years, Bangladesh faces several challenges, including limited and inequitable access to health services, lack of adequate resources to meet the demands of the population and an increasing burden of non-communicable diseases [26–28]. Information and communication technologies, such as District Health Information Software (DHIS), mobile devices to support health systems (mHealth), and telemedicine services can contribute to the improvement of health systems in developing countries [29].

With recommendations from development partners and the World Health Organization (WHO), the Government of Bangladesh implemented a national eHealth policy in 2011 [30, 31]. In July 2011, the Directorate General of Health Services inaugurated the telemedicine service. By 2014, a total of 43 fully-equipped government-operated telemedicine centers were in service [32]. Mobile phone intervention for health is an emerging, rapidly-evolving practice and has been used in improving delivery of health services in many countries of the world [33], including Bangladesh. However, these interventions in Bangladesh mainly focused on child immunization coverage in rural hard-to-reach areas [34] and on attempts to increase adherence to provide for people with other PHC services [35]. To our knowledge, still there is no initiative targeting the rural population to use mobile phones for utilization of primary healthcare and issues of reproductive health as we have chosen for our study.

### Limitations of the study

The study will have a few limitations. A part of the study site has an ongoing health programme of icddr,b, which might have an effect on the intervention. Finally, there is a possibility of recall bias as we will be gathering retrospective information. Lower ability of healthcare providers to use new technology and non-acceptance may affect the project activities. However, we assume that rigorous training and adoption of troubleshooting mechanism can improve the situation.

## Conclusions

Mobile phone-based technology has the potential to improve primary healthcare services, especially those for reproductive health, in low- income countries, like Bangladesh. It is expected that our study will contribute to testing and developing a mobile phone-based intervention to improve the quality and coverage of services. The learning can be used in similar other settings in low- and middle-income countries.

## Additional file

Additional file 1: Household survey questionnaire. (DOCX 91 kb)

## Abbreviations

AHI: Assistant health inspector
ANC: Antenatal care
ARI: Acute respiratory infection
CHCP: Community healthcare provider
CPR: Contraceptive prevalence rate
EATL: Ethics Advance Technology Limited
EPI: Expanded programme on immunization
FP: Family planning
FPI: Family planning inspector
FWV: Family welfare visitor
HA: Health assistant
HI: Health inspector
HPN: Health, population and nutrition
HPNSDP: Health Population and Nutrition Sector Development Programme
MNCH: Maternal, neonatal and child health
MOHFW: Ministry of Health and Family Welfare
PNC: Postnatal care
SACMO: Sub-assistant community medical officer
TFR: Total fertility rate
UFPO: Upazila family planning officer
UH&FPO: Upazila health and family planning officer
UH&FWC: Union health and family welfare centre
