# Supplementary material for: Impact of mobile phone-based technology to improve health, population and nutrition services in Rural Bangladesh: a study protocol
Source: BMC Med Inform Decis Mak. 2017 Jul 6;17:101. doi: 10.1186/s12911-017-0502-9 (PMC5500967; doi:10.1186/s12911-017-0502-9)
Supplement: Additional file 1: — Household survey questionnaire. (DOCX 91 kb) [file 12911_2017_502_MOESM1_ESM.docx]

#

**Impact of mobile phone based technology to improve health, population and nutrition services in rural Bangladesh: A Study protocol**

International Centre for Diarrhoeal Disease Research, Bangladesh (icddr, b)

**Household Survey Questionnaire**

(Respondents are 18-49 years aged married woman and/or a mother having child of 0 to 11 months/12 to 23 months)

**Questionnaire module:**

**Module 01:** Respondent identification

**Module 02:** Information about respondent

**Module 03:** Information about the household and household members

**Module 04:** Mobile phone related knowledge and use

**Module 05:** Knowledge about research programme

**Module 06:** Information about child vaccination

**Module 07:** Information about child health

**Module 08:** Knowledge and practices about pregnancy

**Module 09:** Knowledge and practices about family planning and the methods

**Module 01: Respondent Identifications:**

| 101 | Name of district: |  | |
| --- | --- | --- | --- |
| 102 | Name of upazila: |  | |
| 103 | Name of union: |  | |
| 104 | Name of village: |  | |
| 105 | Name of house: |  | |
| 106 | Serial number of respondent |  | |
| 107 | Name of respondent | ______ | |
| 108 | Husband's name of respondent: | ______ | |
| 109 | Name of interviewer: | _______ | |
| 110 | Date of interview: | \|___\|___\|_____\| | |
| 111 | Starting time of interview | \|___\|___\|:\|___\|___\| | |
| 112 | Respondent Type |  |  |
|  | Mother with 12 to 24 months child | - 1 |  |
|  | Pregnant woman and mother with less than 12 months child | - 2 |  |
|  | Newly married couple | - 3 |  |

**Module 02:** **Information about respondent**

| **Q. No** | **Questions & response** | **Instruction** |
| --- | --- | --- |
| 201 | What is your date of birth?   - \|____\| \|____\| \|______\|   DD MM YYYY   - 99= Don't know | If mention date of birth go to Q. 203 , if answer don't know ask Q. 202 |
| 202 | How old are you?  Please write down in full years   - \|___\|___\| Years |  |
| 203 | What is your educational qualification?  Instruction: If not passed any of the classes then use code 1  SELECT ONE   - 1= No education - 2= Primary incomplete - 3= Primary complete - 4= Secondary incomplete - 5= Secondary complete or higher - 6= Informal education |  |
| 204 | What is your marital status?  SELECT ONE   - 1= Married - 2= Separated - 3= Abandoned - 4= Divorced - 5= Widow | If answer is 1 go to Q. 206, if answer is 2 to 5 ask next Q. |
| 205 | Do you have any child under the age of 24 months?  SELECT ONE ‍   - 1= Yes - 2= No | If answer is “No” stop the interview |
| 206 | What is your occupation?  SELECT ONE   - 1= House wife - 2= Day labour - 3= Govt. service - 4= Private service - 5= Small trader - 6= Business - 7= Student - 8= Others, specify |  |

**Module 03:** **Information about the household and household members**

| **Q. No** | **Questions & response** | | | | | | | **Instruction** |
| --- | --- | --- | --- | --- | --- | --- | --- | --- |
| 301 | I'd like to know about the your child who are under 2 years of age, Please tell the name and date of birth or age from the youngest to the oldest. | | | | | | | If respondent could able to mention the date of birth, then no need to fill up the age box.  If answer of Q. 204 is 2 to 5, skip to Q. 304. |
|  | **No.** | **Name** | **Boy/Girl** | | **Date of birth** | | Age (Month**)** |  |
|  | 1 |  | - Male - Female | | \|___\|___\|_____\| | | \|___\|\|___\| |  |
|  | 2 |  | - Male - Female | | \|___\|___\|_____\| | | \|___\|\|___\| |  |
|  | 3 |  | - Male - Female | | \|___\|___\|_____\| | | \|___\|\|___\| |  |
|  | 4 |  | - Male - Female | | \|___\|___\|_____\| | | \|___\|\|___\| |  |
|  | 5 |  | - Male - Female | | \|___\|___\|_____\| | | \|___\|\|___\| |  |
| 302 | What is your husband's educational qualification?  **Instruction:** If not passed any of the classes then use code 1  SELECT ONE   - 1= No education - 2= Primary incomplete - 3= Primary complete - 4= Secondary incomplete - 5= Secondary complete or higher - 6= Informal education | | | | | | |  |
| 303 | What is the main occupation of your husband?  SELECT ONE   - 1= Farmer - 2= Day labour - 3= Govt. service - 4= Private service - 5= Small business - 6= Business - 7= Rickshaw/Van puller - 8= Auto bike/CNG/Tempo driver - 9= Boat man - 10= Bus/Truck driver - 11= Fisherman - 12= Carpenter - 13= Mason - 14= Garments worker - 15= Unemployed - 16= Other‌, specify__ | | | | | | |  |
| 304 | What is the average of monthly expenditure of the household?   - \|_____________\| Taka | | | | | | |  |
| 305 | What is the average of monthly income of the household?   - \|______________\| Taka | | | | | | |  |
| 306 | Whether the following things are available in your household? | | | | | | | If mobile phone not available in the household, go to next section) |
|  | **Items** | | | **Responses** | | **Numbers** | |  |
|  | A= Mobile phone | | | - 1= Yes - 2= No | | \|___\| | |  |
|  | B= Computer | | | - 1= Yes - 2= No | | \|___\| | |  |
|  | C= Laptop | | | - 1= Yes - 2= No | | \|___\| | |  |
|  | D= Tab | | | - 1= Yes - 2= No | | \|___\| | |  |
|  | E= Television | | | - 1= Yes - 2= No | | \|___\| | |  |
| 307 | To whom the mobile phones are belonging in your household?  Multiple answers acceptable   - A= Self - B= Husband - C= Son - D= Daughter - E= Father-in-law - F= Mother-in-law - G= Brother-in-law - H= Sister-in-law - I= Father - J= Mother - K= Brother - L= Sister - M= There are no mobile phone in this household - N= Others, specify__ | | | | | | |  |

**Module 04:** **Mobile phone related knowledge and use**

| **Q. No** | **Questions & response** | | **Instruction** |
| --- | --- | --- | --- |
| 401 | Now I would like to ask you question about mobile phone. Please tell me, do you use mobile phone?  SELECT ONE   - 1= Yes - 2= No | | If answer is “No” skip to Q.404 |
| 402 | Do you know about the following functions of mobile phone?  Multiple answers acceptable | | If all answer is “Yes” please skip to q. 404 |
|  | **Functions** | **Responses** |  |
|  | A= Make call | - 1= Yes - 2= No |  |
|  | B= Receive call | - 1= Yes - 2= No |  |
|  | C= Understand message alert | - 1= Yes - 2= No |  |
|  | D= Write message | - 1= Yes - 2= No |  |
|  | E= Read message | - 1= Yes - 2= No |  |
|  | F= Send message | - 1= Yes - 2= No |  |
| 403 | In necessary moment how can you ensure the use of mobile phone without knowing it’s any function?  Multiple answers acceptable   - A= With support from family members - B= With support from neighbours - C= With support from relatives - D= No need - E= Other, specify__ | | Please code the correct answer then go to next section |
| 404 | How do you perform mobile based communication without having any mobile phone in your household?  Multiple answers acceptable   - A= Use neighbours mobile - B= Use relative’s mobile - C= Use commercial phone - D= Other, specify__ | |  |

**Module 05: Knowledge about research programme**

| **Q. No** | **Questions & response** | **Instruction** |
| --- | --- | --- |
| 501 | I would like to know about health-related activities provided through mobile phone. Please tell me, is there any service related to maternal, child health and family planning provided in your area through mobile phone?  SELECT ONE   - 1= Yes - 2= No - 3= Don’t know | If answer is “No /Don’t know” go to next session |
| 502 | From whom you have informed about the programme?  Multiple answers acceptable   - A= Govt. health worker - B= NGO health worker - C= icddr,b staff - D= Family member - E= Relatives - F= Neighbours/friends - G= Others, specify__ |  |
| 503 | What programs are undertaking?  Multiple answers acceptable   - A= Newly married couple registration through mobile phone - B= Pregnant women registration through mobile phone - C= Birth notification through mobile phone - D= Newborn registration through mobile phone - E=Inform vaccination schedule to caregivers - F= Inform child health messages to caregivers through mobile phone - G= Inform pregnant mothers on ANC, delivery care and PNC through mobile phone - H= Provide knowledge and motivate for suitable FP method - I= Know the information on field services at offices through electronic devices - J= Able to take necessary measures on field services through electronic devices - K= Others, specify__ |  |
| 504 | When did you know about this programme first time?  SELECT ONE   - 1= Before marriage - 2= After marriage - 3= During pregnancy - 4= During delivery - 5= After delivery - 6= Before vaccination of the child - 7= On vaccination day - 8= After vaccination of the child - 9= Others, specify)__ - 10= Can’t remember |  |
| 505 | Was your name registered by mobile phone?  SELECT ONE   - 1= Yes - 2= No | If answer is “No” go to q. 539 |
| 506 | In which category your name was registered?  Multiple answers acceptable   - 1= MWRA and newly married - 2= Pregnant woman - 3= Mother with less than 24 months child) | If answer is ‘1’ continue the interview, answer is ‘2’, skip to q. 514 and answer is ‘3’ please skip to q. 525 |
| 507 | Did you receive any message/voice call in your mobile phone after registration as newly married couple?  SELECT ONE   - 1= Yes - 2= No | If answer “No” go to Q. 510 |
| 508 | If yes, what was the subject of message/voice call?  Multiple answers acceptable   - A= Inform about suitable family planning method - B= Where and when to go for receiving FP method - C= Inform about the possible side effects of FP methods and remedies - D= Others, specify__ |  |
| 509 | Did you get benefit from the received message/voice call?  SELECT ONE   - 1= Yes - 2= No | If answer is “Yes” skip to q. 511 |
| 510 | If no, what was the reason for not getting the message/voice call as you think?  Multiple answers acceptable   - A= Mobile set was out of order/lost - B= Changing SIM - C= Don’t know - D= Others, specify__ |  |
| 511 | What is your opinion about this health care service delivery process through mobile phone?  SELECT ONE   - 1= Excellent - 2= Good - 3= Moderate - 4= Not good - 5= Bad - 6= No response | If answer is 3 to 5, go to Q. 513 and answer is 6 please go to q. 539 |
| 512 | If the answer is excellent/good, what is the reason behind this?  Multiple answers acceptable   - A= Can get Family Planning related information at home - B= Saves time & don’t hamper work schedule - C= Do not need travel cost - D= Others, specify__ | Select exact code then go to Q. 539 |
| 513 | If the answer is not good/ bad/moderate, what is the reason behind this?  Multiple answers acceptable   - A= Difficult to read message - B= Difficult to hear voice calls - C= Need to have own mobile phone - D= Problem occurs while mobile phone is out of order/lost - E= Problem occurs if SIM changes - F= Breach of privacy - G= Others, specify__ | Select exact code then go to Q. 539 |
| 514 | As a pregnant woman, did you receive any message/voice call through mobile phone?  SELECT ONE   - 1= Yes - 2= No | If answer “No” go to Q. 520 |
| 515 | If yes, what message/voice call did you receive?  Multiple answers acceptable   - A= Information about ANC - B= Information about delivery care - C= Information about PNC - D= Others, specify__ |  |
| 516 | Did you get benefit from that message/voice call?  SELECT ONE   - 1= Yes - 2= No |  |
| 517 | What is your opinion about health care service delivery process through mobile phone?  SELECT ONE   - 1= Excellent - 2= Good - 3= Moderate - 4= Not good - 5= Bad - 6= No response | If answer is 3 to 6 go to Q. 519 and if and answer is 6 please go to q. 539 |
| 518 | If the answer is excellent/good, what is the reason behind this?  Multiple answers acceptable   - A= Can get ANC and PNC related information at home - B= Saves time & don’t hamper work schedule - C= Do not need travel cost - D= Others, specify__ | Select exact code then go to Q. 520 |
| 519 | If the answer is not good/ bad/moderate, what is the reason behind this?  Multiple answers acceptable   - A= Difficult to read message - B= Difficult to hear voice calls - C= Need to have own mobile phone - D= Problem occurs while mobile phone is out of order/lost - E= Problem occurs if SIM change/lost - F= Breach of privacy - G= Others, specify__ |  |
| 520 | Instructions: Only applicable for 0-11 month child’s mother.  Did you/on behalf of you notify the birth of newborn to the specific number?  SELECT ONE   - 1= Yes - 2= No - 3= Not applicable | If answer is “No” go to G. 522 and answer is N/A, go to q. 525 |
| 521 | If no, why not sent?  Multiple answers acceptable   - A= Lost the number - B= Did not have own mobile phone - C= Forget to send the message - D= Did not think as necessary - E= Do not know that message has to be sent - F= The process of sending message is complex - G= Network problem - H= No credit in mobile phone - I= Tried but not succeed - J= Others, specify__ |  |
| 522 | What is your opinion about this health care service delivery process through mobile phone?)  SELECT ONE   - 1= Excellent - 2= Good - 3= Moderate - 4= Not good - 5= Bad - 6= No response | If answer is 3 to 5 go to Q. 524 and answer is 6 please go to q. 525 |
| 523 | If the answer is excellent/good, what is the reason behind this?  Multiple answers acceptable   - A= Ensure accurate date of birth of the child - B= Get child’s vaccination related information at home - C= The child is not left out/drop out from vaccination as he/she registered - D= Saves time & don’t hamper work schedule - E=Do not require travel cost - F= Others, specify | Select exact code then go to Q. 525 |
| 524 | If the answer is not good/ bad/ moderate, what is the reason behind this?  Multiple answers acceptable   - A= Require own phone - B= The process of sending message is complex - C= Can’t remember to send message - D= Nobody helped - E= Network problem - F= Need to remain credit in mobile phone - G= Others, specify__ |  |
| 525 | Did you receive any message/voice call on child health?  SELECT ONE   - 1= Yes - 2= No | If answer is “No” go to Q. 528 |
| 526 | If yes, what message/voice call did you receive?  Multiple answers acceptable   - A= Child health related information - B= Where to go during child illness - C= Others specify__ |  |
| 527 | Did you get benefit from that message/voice call?  SELECT ONE   - 1= Yes - 2= No | Select right answer then please go to Q. 529 |
| 528 | If no, Why did not you think?  Multiple answers acceptable   - A= Mobile set was out of order/lost) - B= Change SIM - C= Don’t know - D= Not applicable - E= Others, specify__ |  |
| 529 | What is your opinion about this health care service delivery process through mobile phone?  SELECT ONE   - 1= Excellent - 2= Good - 3= Moderate - 4= Not good - 5= Bad - 6= No response | If answer is 3 to 5 go to Q. 531 and answer is 6 please go to q. 532 |
| 530 | If the answer is excellent/good, what is the reason behind this?  Multiple answers acceptable   - A= Can get child health related information at home - B= Saves time & don’t hamper work schedule - C= Do not need travel cost - D= Others, specify__ | Select exact code then go to Q. 532 |
| 531 | If the answer is not good/ bad/ moderate, what is the reason behind this?  Multiple answers acceptable   - A= Difficult to read message - B= Difficult to hear voice calls - C= Need to have own mobile phone - D= Problem occurs while mobile phone is out of order/lost - E=Problem occurs if SIM change/lost - F= Others, specify__ - G= Not applicable |  |
| 532 | Did you receive any message/voice call on child vaccination?  SELECT ONE   - 1= Yes - 2= No | If answer is “No”go to Q. 535 |
| 533 | If yes, what message/voice call did you receive?  Multiple answers acceptable   - A= Message/Voice call on child vaccination information - B=Child vaccination schedule - C= Reminder on going to vaccination centre - D= Where to go during child illness - E= Others specify__ |  |
| 534 | Did you get benefit from that message/voice call?  SELECT ONE   - 1= Yes - 2= No | Select exact code then go to Q. 536 |
| 535 | If no, what do you think for not receiving the message/voice call?  Multiple answers acceptable   - A=Mobile set was out of order/lost - B= Changing SIM - C= Don’t know - D= Others, specify__ - E= Not applicable |  |
| 536 | What is your opinion about this health care service delivery process through mobile phone?  SELECT ONE   - 1= Excellent - 2= Good - 3= Moderate - 4= Not good - 5= Bad - 6= No response | If answer is 3 to 6 no go to Q. 538 |
| 537 | If the answer is excellent/good, what is the reason behind this?  Multiple answers acceptable   - A= Can get child vaccination related information at home - B= Save time & don’t hamper work schedule - C= Do not need travel cost - D= Others, specify)__ | Select exact code then go to Q. 539 |
| 538 | If the answer is not good/ bad/ moderate, what is the reason behind this?  Multiple answers acceptable   - A= Difficult to read message - B= Difficult to hear voice calls - C= Need to have own mobile phone - D= Problem occurs while mobile phone is out of order/lost - E= Problem occurs if SIM change/lost - F =Others, specify__ - G= Not applicable |  |
| 539 | What is your opinion about mobile based monitoring process?  SELECT ONE   - 1= Excellent - 2= Good - 3= Moderate - 4= Not good - 5= Bad - 6= No response | If answer is 3 to 5 go to Q. 541 and answer is 6 please go to q. Next section |
| 540 | If the answer is excellent/good, what is the reason behind this?  Multiple answers acceptable   - A= Increase accountability - B= Can provide quick feedback - C= Increase quality of work - D= Reduces fault in work - E= Easy to undertake next step - F= Others, specify__ | Select exact code then go to Q. 601 |
| 541 | If the answer is not good/ bad/ moderate, what is the reason behind this?  Multiple answers acceptable   - A= Require Laptop/computer - B= Require internet connection - C= Require education - D= Require technical knowledge - E= Others, specify__ - F= Not applicable |  |

**Module 06:** **Information about child vaccination**

**Instructions:** This section is applicable only for 12 to 23 months aged children

| **Q. No.** | **Questions & response** | | | | | **Instruction** |
| --- | --- | --- | --- | --- | --- | --- |
| 601 | Now I would like to ask you about the vaccination status of your child (Child Name). Would you please tell whether the child has received any disease preventive vaccine under routine vaccination programme?)  SELECT ONE   - 1= Yes - 2= No | | | | | If answer is “Yes” go to Q. 603 |
| 602 | If not, what is the reason for not getting the vaccine?  Multiple answers acceptable   - A= Fear of side effect - B= Do not have faith on vaccination - C= Due to child illness - D= Didn't know that vaccine should be given - E= Due to business - F= Due to own illness - G= Due to child’s cry - H= Thought, to be vaccinated later - I= Can't remember - J= Thought the vaccinator would come to home - K= Needs money for vaccination - L= Husband did not permit - M= Don't know where to go for vaccination - N=Visited outside - O= Father-in-law/mother-in-law did not permit - P= Vaccination centre is too far - Q= Don’t receive any message/information through mobile - R= Don’t understand the message - S= Don’t know the vaccination session schedule - T= Other, specify | | | | |  |
| 603 | Do you have any suggestion to vaccinate all target children in your area?  SELECT ONE   - 1= Yes - 2= No | | | | | If answer is “No” go to Q. 605 |
| 604 | What are the suggestions?  Multiple answers acceptable   - A=All pregnant women need to be registered through mobile phone - B= Birth information of all neonates need to be registered through mobile phone - C= Mothers should be informed day before the vaccination schedule date through mobile phone - D= Mothers should be informed on the vaccination day through mobile phone - E= Spreading out the importance of vaccination - F= Left out children should be searched more carefully - G= Should be informed before the session - H= Should be informed through announcement - I= Vaccinator should visit door to door for vaccination - J= Vaccination should be free of cost - K= Mother should be informed door to door day before the vaccination day - L=Others, specify | | | | | Select the exact answer of q. 604 if no go to next section |
| 605 | What is the status of the child's vaccination card?  SELECT ONE   - 1= Card received and available - 2= Card received but not available - 3= Never received card - 4= Don't know - 5= Not applicable | | | | | If answer is 2-4, go to Q. 610 if answer NA go to 701 |
| 606 | Can you show me the card?  SELECT ONE   - 1= Yes - 2= No | | | | | If answer is “Yes” go to Q.608 |
| 607 | If no, where is the card now?  SELECT ONE   - 1= Locked at almirrah - 2= The person is not at home who preserve the card - 3= Card is lost - 4= Card is in another house - 5= Other, Specify__ | | | | |  |
| 608 | Has your child received any vaccine that is not recorded on the card?  SELECT ONE   - 1= Yes - 2= No - 3= Don't know | | | | | If answer is “No or Don’t know” then go to Q. 610 |
| 609 | If yes, why not recorded on the card?  Multiple answers acceptable   - A= Guardian did not bring the card in the EPI centre - B= By mistake, the vaccinator did not write in the card - C= Vaccine was received from different centre - D= Vaccinator put tick mark without mentioning the date on the card - E= Do not know the reason - F= Lost the previous card - G= Others, specify__ | | | | |  |
| 610 | Did your child ever return from vaccination centre without vaccination?  SELECT ONE   - 1= Yes - 2= No - 3= Don't know | | | | | If answer is “No or Don’t know” go to Q. 612 |
| 611 | If yes, what happened?  Multiple answers acceptable   - A= Child name was not registered - B= Not having vaccination card - C= Advised to go in the next day with card - D= Demand money - E= Due to misbehave - F= Child was sick - G= Not going to scheduled vaccination centre - H= Went after the session was over - I= The vaccination centre was crowded - I= Others, specify__ | | | | |  |
| 612 | Enter the date of birth of the child according to the card   - \|__\|__\|____\| - 88 = Card not available)   **Instructions:** Write down the vaccination date according to the card. If the date is not written or tick marked in the vaccination card, please put “0” in the box and if the date is illegible, please put “9” | | | | | If card not available ‍skip to Q. 614 |
| 613 | Enter the date according to the vaccination card | | | | | Write exact date then go to Q. 616 |
|  | - A= BCG | | | \|___\|___\|____\| | |  |
|  | - B= PENTA-1 | | | \|___\|___\|____\| | |  |
|  | - C= PENTA-2 | | | \|___\|___\|____\| | |  |
|  | - D= PENTA-3 | | | \|___\|___\|____\| | |  |
|  | - E= PCV-1 | | | \|___\|___\|____\| | |  |
|  | - F= PCV-2 | | | \|___\|___\|____\| | |  |
|  | - G= PCV-3 | | | \|___\|___\|____\| | |  |
|  | - H= OPV-1 | | | \|___\|___\|____\| | |  |
|  | - I= OPV-2 | | | \|___\|___\|____\| | |  |
|  | - J= (OPV-3 | | | \|___\|___\|____\| | |  |
|  | - K= IPV | | | \|___\|___\|____\| | |  |
|  | - L= MR | | | \|___\|___\|____\| | |  |
|  | - M= MSD/MR - 2nd Dose | | | \|___\|___\|____\| | |  |
| 614 | **Instructions:** If card is not available, then fill up question no 615 boxes as per respondent statement. Please ensure the vaccination information by asking following questions before fill up the boxes. | | | | |  |
|  | **Visit no** | **Number of vaccine** | **Which part of the body** | | **Age of child** |  |
|  | First time | ______ | - A= Mouth - B= Left arm - C= Right thigh - D= Left thigh | | ______ |  |
|  | Second time | ______ | - A= Mouth - B=Left arm - C= Right thigh - D= Left thigh | | ______ |  |
|  | Third time | ______ | - 1= Mouth - 2= Left arm - 3= Right thigh - 4= Left thigh | | ______ |  |
|  | Fourth time | ______ | - A= Mouth - B= Left arm - C= Right thigh - D= Left thigh | | ______ |  |
|  | Fifth time | ______ | - A= Mouth - B= Left arm - C= Right thigh - D= Left thigh | | ______ |  |
|  | Sixth Time | ______ | - A= Mouth - B= Left arm - C= Right thigh - D= Left thigh | | ______ |  |
| 615 | Fill this questions according to q. 514 | | | | |  |
|  | - A= BCG | | | | |  |
|  | - B= PENTA-1 | | | | |  |
|  | - C= PENTA-2 | | | | |  |
|  | - D= PENTA-3 | | | | |  |
|  | - E= PCV-1 | | | | |  |
|  | - F= PCV-2 | | | | |  |
|  | - G= PCV-3 | | | | |  |
|  | - H= OPV-1 | | | | |  |
|  | - I= OPV-2 | | | | |  |
|  | - J= (OPV-3 | | | | |  |
|  | - K= IPV | | | | |  |
|  | - L= MR | | | | |  |
|  | - M= MSD/MR - 2nd Dose | | | | |  |
| 616 | **Instructions:** If the child not get all vaccines (up to MR), then ask the bellow question  What were the reasons for not giving all doses of vaccines to your child?  Multiple answers acceptable   - A= Fear of side effect - B= Not having faith on vaccination - C= Due to child illness - D= Didn't know that vaccine should be given - E= Due to business - F= Due to own illness - G= Due to child’s cry - H= Thought, to be vaccinated later - I= Can't remember - J= Thought the vaccinator would come to home - K= Needs money for vaccination - L= Husband did not permit - M= Don't know where to go for vaccination - N= Visited outside - O= Father-in-law/mother-in-law did not permit - P= Vaccination centre is too far - Q= Don’t receive any message/information through mobile - R= Don’t understand the message - S= Don’t know the vaccination session schedule - T= Not applicable - U= Other, specify_ | | | | |  |
| 617 | Do you have any suggestion to vaccinate all the children of your area with all doses of vaccines?  SELECT ONE   - 1= Yes - 2= No | | | | | If answer is “No” go to Q. 701 |
| 618 | What are the suggestions?  Multiple answers acceptable   - A= All pregnant women need to be registered through mobile phone - B= Birth information of all neonates need to be registered through mobile phone - C= Mothers should be informed day before the vaccination schedule date through mobile phone - D= Mothers should be informed on the vaccination day through mobile phone - E= Spreading out the importance of vaccination - F= Left out children should be searched more carefully - G= Should be informed before the session - H= Should announce through mike - I= Vaccinator should visit door to door for vaccination - J=Vaccination should be free of cost - K= Others, specify__ | | | | |  |

**Module 07:** **Information about child health**

**Instructions**: This section is applicable only for 12 to 23 months aged children

| **Q. No.** | **Questions & response** | **Instruction** |
| --- | --- | --- |
| 701 | Was the child aged 12 to 23 months of your households taken to a doctor or to a hospital/ clinic in the last 3 months due to illness?  SELECT ONE   - 1= Yes - 2= No | If answer is“No” go to Q. 801 |
| 702 | If yes, for what disease?  Multiple answers acceptable   - A= Pneumonia - B= Dysentery - C= Diarrhoea - D= Ear infection - E= Measles - F= Malaria - G= Measles (Eye and oral complication - H= Pallor - I= Low weight - J= Feeding problem - K= No pneumonia- wheeze - L= Others, specify__ |  |
| 703 | Where the child was taken for treatment?  Multiple answers acceptable   - A= Pharmacy - B= Doctor’s chamber - C= Satellite clinic/EPI Centre - D= Community clinic - E= Maternity and child welfare centre - F= Upazila health complex - G= District/sador hospital - H= Medical college hospital - I= Other public health centre - J= NGO hospital/clinic - K= Private hospital/clinic - L= Doctor visited the patient at home - M= Others, specify__ |  |
| 704 | Who motivated you for child’s treatment?  Multiple answers acceptable   - A= Family member - B= Relative - C= Neighbor/friend - D= Health worker - E= Village doctor - F= SMS through mobile phone - G= Voice call - H= Self initiative - I= Others, specify__ |  |

**Module 08:** **Knowledge and practices about pregnancy**

Instructions: this section is applicable only pregnant woman

| **(Q. No.** | **Questions & response** | **Instruction** |
| --- | --- | --- |
|  | **Antenatal care:** |  |
| 801 | Have you ever heard about antenatal care?  SELECT ONE   - 1= Yes - 2= No | If answer “No” go to question no 806 |
| 802 | If yes, what have you heard?  Multiple answers acceptable   - A= Needs regular check up - B= Needs TT vaccination - C= Should take iron tablet) - D= Needs to visit the doctor at hospital for check-up - E= Needs measuring the weight - F= Needs for doing ultra sonogram - G= Should take nutritious food - H= Restricted to lift heavy items - I= Should take adequate rest - J= Needs frequent meal - K= Others, specify__ |  |
| 803 | Can you tell me how many times the antenatal check-up should be performed?  SELECT ONE   - 1= One time - 2= Two times - 3= Three times - 4= Four times - 5= Don’t know - 6= Others, specify__ |  |
| 804 | Can you tell who does provide the antenatal check-up?  Multiple answers acceptable   - A= Community Health Care Provider - B= Family Welfare Assistant - C= Health Assistant - D= Family Welfare Visitor - E= SACMO/paramedic - F= MBBS doctor - G= NGO health worker - H= Others, specify__ |  |
| 805 | Do you know where you can get antenatal services?  (Multiple answers acceptable)   - A= Community Clinic - B= Satellite Clinic - C= UH&FWC - D= UHC - E= District hospital/sador hospital - F= MCWC - G= Medical college hospital - H= NGO hospital - I= Private clinic - J= Doctor’s chamber - K= Pharmacy/village doctor - L= Others, specify__ |  |
| 806 | Have you ever heard about the dangers signs of pregnancy?)  SELECT ONE   - 1= Yes - 2= No | If answer “No” go to question no 809 |
| 807 | What are the danger signs of pregnancy?  Multiple answers acceptable   - A= Bleeding - B= Headache/blurred vision - C= Severe fever - D= Severe abdomen pain - E= Convulsion - F= Oedema in hands and legs - G= Others, specify__ |  |
| 808 | From whom you have heard this information?  Multiple answers acceptable   - A= Family member - B= Relative - C= Neighbour - D= Health worker - E= MBBS doctor - F= TV/radio - G= Newspaper - H= Bill board - I= SMS through mobile phone - J= Video call - K= Others, specify__ |  |
| **Practice about antenatal:** | |  |
| 809 | Are you currently pregnant?  SELECT ONE   - 1= Yes - 2= No | If answer “No” go to question no 814 |
| 810 | If yes, what is the gestational age?  SELECT ONE   - 1= <3 months - 2= 4 to 6 months - 3= 7 to 9 months - 4= Above 9 months - 5= Cannot say |  |
| 811 | Did you perform antenatal check-up?  SELECT ONE   - 1= Yes - 2= No | If answer “No” go to question no 813 |
| 812 | How many times did you perform antenatal check-up?  SELECT ONE   - 1= One time - 2= Two times - 3= Three times - 4= Four times - 5= Can’t remember - 6= Others, specify__ | Please write the right answer then go to question no 814 |
| 813 | If no, why?  Multiple answers acceptable   - A= Did not feel as necessary - B= As did not checkups in previous pregnancies - C= Did not have money - D= Restriction from family - E= Husband did not allow - F= As there was no problem - G= Don’t receive any message/information through mobile - H= Don’t understand the message - I= Others, specify_ |  |
| 814 | Have you performed antenatal check-up during last pregnancy?  SELECT ONE   - 1= Yes - 2= No - 3= Not applicable | If answer “No or NA” go to question no 816 |
| 815 | If no, why?  Multiple answers acceptable)   - A= Did not feel as necessary - B= As did not check up in previous pregnancies - C= Did not have money - D= Restriction from family - E= Husband did not allow - F= As there was no problem - G= Spontaneous abortion/miscarriage - H= Did not receive any message/information through mobile - I= Don’t understand the message - J= Others, specify | Please write the right answer then go to question no 817 |
| 816 | How many times did you perform ante natal check-up during last pregnancy  SELECT ONE   - 1= One time) - 2= Two times) - 3= Three times) - 4= Four times) - 5= Cannot remember - 6Others, specify__ |  |
| 817 | Did you have any complication during your last pregnancy?  SELECT ONE   - 1= Yes - 2= No | If answer “No” go to question no 822 |
| 818 | Did you visit any doctor for these complications?  SELECT ONE   - 1= Yes - 2= No | If answer “No” go to question no 821 |
| 819 | Where did you visit the doctor?  Multiple answers acceptable   - A= CC - B= SC - C= UH&FWC - D= UHC - E= District hospital/sador hospital - F= MCWC - G= Medical college hospital - H= NGO hospital - I= Private clinic - J= Doctor’s chamber - K= Pharmacy/Village doctor - L= Others, specify__ |  |
| 820 | Who advised you to visit doctor?  Multiple answers acceptable   - A= Family member - B= Relatives - C= Neighbour - D= Health worker - E= MBBS doctor - F= TV/radio - G= Newspaper - H= Bill board - I= SMS through mobile - J= Voice call - K= Self initiative - L = Others, specify__ |  |
| 821 | If no, why?  Multiple answers acceptable   - A= Did not give importance - B= Taken medicine from pharmacy - C= Get cured  spontaneously - D= Did not have money - E= Barrier from family - F= Husband did not permit - G= Others, specify__ |  |
| **Knowledge about postnatal care:** | |  |
| 822 | Have you ever heard about postnatal care?  SELECT ONE   - 1= Yes - 2= No | **If answer is No, go to Q. 825** |
| 823 | What have you heard about postnatal care?  Multiple answers acceptable   - A= Mother should take vitamin A capsule within 42 days of delivery - B= Should check up by doctor within 24 hours of delivery - C= Mother and child should be kept warm - D= Mother should take more water and nutritious food after delivery - E= Keep mother in mental peace - F= Mother should visit the doctor three times after the delivery - G= Others, specify__ |  |
| 824 | Can you tell me how many times you should perform post natal check up /visit the doctor?  SELECT ONE   - 1= One time - 2= Two times - 3= Three times - 4= Four times - 5= Do not remember - 6= Others, specify__ |  |
| 825 | Have you ever hard about the post-partum danger?  SELECT ONE   - 1= Yes - 2= No | If answer is No, go to Q. 829 |
| 826 | What you have heard post-partum danger sign?  Multiple answers acceptable)   - A= Bleeding - B= Headache/blurred in vision - C= High fever - D= Severe abdominal pain - E= Convulsion - F= Oedema hands and legs - G= Others, specify__ |  |
| 827 | Do you know where to go after appearance of the post-partum danger sign?  Multiple answers acceptable   - A= CC - B= SC - C= UH&FWC - D= UHC - E= District hospital/sador hospital - F= MCWC - G= Medical college hospital - H= NGO hospital - I= Private clinic - J= Private doctor’s chamber - K= Pharmacy/Village doctor) - L= Others, specify__ |  |
| 828 | From whom you have heard this information?  (Multiple answers acceptable)   - A= Family member - B= Relative - C= Neighbour - D= Health worker - E= MBBS doctor - F= TV/radio - G= Newspaper - H= Bill board - I= SMS through mobile - J= Voice call - K= Others, specify__ | Check 814 if answer 3 then go q101 |
| **Experiences about postnatal care:** | |  |
| 829 | How many times did you perform post natal check-up?  SELECT ONE   - 1= One time - 2= Two times - 3= Three times or more - 4= Do not remember - 5= Never - 6= Others, specify__ |  |
| 830 | Where was your last delivery occurred?  SELECT ONE   - 1= Husband house - 2= Father’s house - 3= UH&FWC - 4= UHC - 5= District hospital/sador hospital - 6= MCWC - 7= Medical college hospital - 8= NGO hospital - 9= Private clinic - 10= Others, specify__ |  |
| 831 | Did you feed colostrums your newborn  SELECT ONE   - 1= Yes - 2= No - 3= Not applicable | If answer is Yes and Not applicable then go to Q. 833 |
| 832 | If no, why did you not feed colostrums to the newborn?  (Multiple answers acceptable)   - A= As rotten milk - B= No milk in breast - C= Nobody informed about the feeding of the colostrums - D= Did not know the benefits of colostrums - E= Elders discouraged to feed colostrums - F= Others, specify__ |  |
| 833 | Did you take vitamin A capsule within 42 days of delivery?  SELECT ONE   - 1= Yes - 2= No - 3= Not applicable |  |
| 834 | Was there any complication during your last delivery  SELECT ONE   - 1= Yes - 2= No | If answer is No, go to Q.1001 |
| 835 | If yes, what were the complications?  (Multiple answers acceptable)   - A= Profuse bleeding after delivery - B= Severe headache/blurred vision - C= High fever - D= Hand/feet prolapsed - E= Convulsions - F= Labour pain lasted more than twelve hours - G= Others, specify__ |  |
| 836 | Did you visit any doctor for these complications?  SELECT ONE   - 1= Yes - 2= No | If answer is No, go to Q. 839 |
| 837 | Where did you see the doctor?  (Multiple answers acceptable)   - A= CC - B= SC - C= UH&FWC - D= UHC - E= District hospital/sador hospital - F= MCWC - G= Medical college hospital - H= NGO hospital - I= Private clinic - J= Private chamber - K= Pharmacy/Village doctor - L= Others, specify__ |  |
| 838 | Who advised you to visit doctor?  (Multiple answers acceptable)   - A= Family member) - B= Relative - C= Neighbour - D= Health worker - E= MBBS doctor) - F= TV/radio - G= Newspaper - H= Bill board) - I= SMS through mobile - J= Video call - K= Others, specify__ | Code correct answer then skip to q. 1001) |
| 839 | If no, why not?  Multiple answers acceptable   - A= Did not give importance - B= Taken medicine from pharmacy - C= Get cured  spontaneously - D= Did not have money - E= Restriction from family - F= Husband did not permit - G= Others, specify__ - H= Not applicable |  |

**Module 09:** **Knowledge and practices about family planning and the methods**

| **(Q. No.** | **Questions & response** | **Instruction** |
| --- | --- | --- |
| 901 | Have you ever heard any method of contraception by which a couple can delay or stop the pregnancy?)  SELECT ONE   - 1= Yes - 2= No | (If answer is No, end the Interview) |
| 902 | If yes, what are the methods?  (Multiple answers acceptable)   - A= Female sterilization - B= Male sterilization - C= Oral pill - D= IUD - E= Injection depo-provera - F= Implant/norplant - G= Condom - H= Safe period - I= Abstain - J= Withdrawal - K= Lactational amenorrhoea method (LAM) - L= Others, specify__ |  |
| 903 | Multiple answers acceptable   - A= FWA - B= CHCP - C= HA - D= FWV - E= Nurse - F= Paramedics - G= Medical Assistant/SACMO - H= MBBS doctor - I= CSBA - J= TBA - K= Village doctor - L= Drug seller - M= Relatives - N= Through mobile phone message - O= Voice call - P= Billboard/radio/TV - Q= Others, specify__ |  |
| 904 | Have you/your husband currently use any method?  SELECT ONE   - 1= Yes - 2= No | If answer is No, go to Q. 906 |
| 905 | If yes, what are these methods?  Multiple answers acceptable   - A= Female sterilization - B= Male sterilization - C= Oral pill - D= IUD - E= Injection Depo-Provera - F= Implant/Norplant - G= Condom - H= Safe period - I= Abstain - J= Withdrawal - K= Lactational amenorrhea method (LAM) - L= Others, specify__ | Code correct answer then skip to q. 907 |
| 906 | What are the reasons for not using any method?  Multiple answers acceptable   - A= Infrequent sex/no sex - B= Onset of menopause/ Hysterectomy - C= Unable to bear children - D= Wants as many children as possible - E= oppose to use - F= Respondents opposed - G= Husband does not like - H= Others opposed - I= Religious barriers - J= Lack of knowledge) - K= Adverse effects on breast milk - L= Knows no source - M= Health concerns - N= Fear of side effects - O= Lack of access/too far - P= Costs too much - Q= Inconvenient to use - R= Interfere with normal physiological process= - S= Other (Specify)__ - T= Don't know - U= Not applicable | Select exact code and go to 1001 |
| 907 | Who advised you to use contraceptive?  (Multiple answers acceptable)   - A= FWA - B= HA - C= FWVD= Nurse - E= Paramedics - F= Medical Assistant/SACMO - G= MBBS doctor - H= TTBA - I= UTBA - J= Village doctor - K= Drug seller - L= Relatives - M= SMS through mobile - N= Voice call - O= Self decision - P= Others, specify)__ |  |
| 908 | At present, are you or your husband facing any side effect(s) for using the contraceptive methods?)  SELECT ONE   - 1= Yes - 2= No | If answer is No, go to Q 1001. |
| 909 | If yes, what are the side effects?  (Multiple answers acceptable)   - A= Gaining weight - B= Weight loosing - C= Excessive bleeding - D= High blood pressure - E= Headache - F= Dizziness - G= Excessive ministration - H= Weakness/illness/tiredness - I= Abdominal pain - J= White discharge - K= Complicacy during menstruation - L= Decrease breast milk - M= Nausea - N= Restlessness - O= Others, specify__ |  |
| 910 | Did you discuss with anybody when you faced these side effects?  SELECT ONE   - 1= Yes - 2= No | If answer is then go to 1001) |
| 911 | With whom you discussed about these side effects?  Multiple answers acceptable   - A= MBBS doctor - B= FWV - C= FWA - D= HA - E= SACMO/Medical Assistant - F= Nurse - G= Paramedics - H= NGO depot holder - I= NGO field worker - J= Village doctor - K= Pharmacist - L= Relatives/friends - M= Husband/wife - N= Nobody/own self - O= Homeopathy doctor - P= Others, specify__ |  |
| 912 | He/they advised to visit a doctor?  SELECT ONE   - 1= Yes - 2= No | If answer is then go to 1001 |
| 913 | Who advised you to visit doctor?  (Multiple answers acceptable)   - A= Family members - B= Relatives - C= Neighbour - D= Health worker - E= MBBS doctor - F= TV/radio - G= Newspaper - H= Bill board - I= Message through mobile - J= Video call - K= Self initiative - L= Others, specify__ |  |

| 1001 | Ending time of interview | \|___\|___\|:\|___\|___\| |
| --- | --- | --- |
| 1002 | Status of interview: | - 1= Completed - 2= In completed |

Thank you for participating in the interview
